# A rare cause of epileptic encephalopathy: case report of a novel patient with PEHO-like phenotype and *CCDC88A* gene pathogenic variants

**DOI:** 10.1186/s13052-024-01766-y

**Published:** 2024-09-27

**Authors:** Sorina-Mihaela Papuc, Adelina Glangher, Alina Erbescu, Oana Tarta Arsene, Aurora Arghir, Magdalena Budisteanu

**Affiliations:** 1 Medical Genetics Laboratory, Victor Babes National Institute of Pathology, Bucharest, 050096 Romania; 2 Psychiatry Research Laboratory, Prof. Dr. Alex. Obregia Clinical Hospital of Psychiatry, Bucharest, 041914 Romania; 3 Pediatric Neurology Department, Prof. Dr. Alex. Obregia Clinical Hospital of Psychiatry, Bucharest, 041914 Romania; 4 Faculty of Medicine, Department of Genetics, Titu Maiorescu University, Bucharest, 031593 Romania

**Keywords:** Epileptic encephalopathy, Cortical brain malformations, Novel biallelic variants, Global developmental delay

## Abstract

**Background:**

The Coiled-Coil Domain-Containing Protein 88 A (*CCDC88A*) gene encodes the actin-binding protein Girdin, which plays important roles in maintaining the actin cytoskeleton and in cell migration and was recently associated with a specific form of epileptic encephalopathy. Biallelic protein-truncating variants of *CCDC88A* have been considered responsible for progressive encephalopathy with edema, hypsarrhythmia, and optic atrophy (PEHO)-like syndrome. To date, only three consanguineous families with loss-of-function homozygous variants in the *CCDC88A* gene have been reported. The described patients share many clinical features, such as microcephaly, neonatal hypotonia, seizures, profound developmental delay, face and limb edema, and dysmorphic features, with a similar appearance of the eyes, nose, mouth, and fingers.

**Case presentation:**

We report on a child from a nonconsanguineous family who presented with profound global developmental delay, severe epilepsy, and brain malformations, including subcortical band heterotopia. The patient harbored two heterozygous pathogenic variants in the *trans* configuration in the *CCDC88A* gene, which affected the coiled-coil and C-terminal domains.

**Conclusions:**

We detail the clinical and cerebral imaging data of our patient in the context of previously reported patients with disease-causing variants in the *CCDC88A* gene, emphasizing the common phenotypes, including cortical malformations, that warrant screening for sequence variants in this gene.

## Background

Epileptic encephalopathies (EEs) are rare but complex conditions with poor outcomes, given their association with epileptic seizures and psychomotor development. Most of these conditions have a genetic cause, and advances in next generation sequencing (NGS) technologies have greatly contributed to the identification of many genes involved in EE pathogenesis. One of the genes recently associated with EE is the Coiled-Coil Domain-Containing Protein 88 A (CCDC88A) gene. The *CCDC88A* gene encodes the actin-binding protein Girdin, which is expressed in fetal and adult tissues, including the brain [4, 17]. The actin-binding protein Girdin plays an important role in both maintaining the actin cytoskeleton and in cell migration [4]. Biallelic protein-truncating variants of *CCDC88A* have been considered responsible for progressive encephalopathy with edema, hypsarrhythmia, and optic atrophy (PEHO)-like syndrome (MIM 617507), based on the clinical and molecular data of the first three children ever reported with this condition [18]. Specifically, Nahorski et al. [18] investigated three children from a single family who presented with epileptic encephalopathy, hypotonia, brain malformations, and microcephaly, with severe disease evolution (i.e., drug-resistant epilepsy and profound global developmental delay). In another study, Abdulkareem et al. (2018) reported two siblings from a Saudi family with epilepsy and developmental delay. Recently, Issa et al. [10] described a girl from an Egyptian family with seizures, global developmental delay, progressive microcephaly, and poor visual responsiveness. These children, from three consanguineous families, are the only patients with PEHO/PEHO-like syndrome harboring *CCDC88A* pathogenic variants reported to date. The *CCDC88A* variants of these patients were homozygous, protein-truncating, and located in the coiled-coil domain of the protein.

Here, we report the case of a new patient, a two-year 10-month-old boy from a nonconsanguineous family, who presented with profound global developmental delay, severe epilepsy, and brain malformations, including subcortical brain heterotopia. The patient harbored a compound heterozygous pathogenic variant in the *CCDC88A* gene that affected the coiled-coil and C-terminal domains. We detail the clinical and brain imaging data of our patient in the context of previously reported patients with disease-causing variants in the *CCDC88A* gene.

## Case presentation

### Clinical evaluation

Our patient, a two-year 10-month-old boy, was born of nonconsanguineous parentage after a full-term pregnancy with a birth weight of 3120 g, an Apgar score of 9, and good postnatal adaptation.

The patient was referred to our clinic for epileptic seizures with neonatal onset and global developmental delay. A general clinical evaluation consisting of a neurologic and dysmorphological examination was performed at admission. The child underwent awake and sleep electroencephalography (EEG) studies and 1.5T brain magnetic resonance imaging (MRI), which was performed according to the standard protocols.

Prenatal brain ultrasound performed at 29 weeks of gestation showed ventriculomegaly. At birth, the following dysmorphic facial features were noted: hypertelorism, wide nasal root, micro retrognathia, short neck, micro-pupils, and microcephaly. Seizure onset was within the first hour of life, with a polymorphic aspect categorized into three types: a first type with bilateral hypertonia, skin hyperemia, disorganized eye movements, and masticatory automatisms; a second type with disorganized eye movements and masticatory automatisms; and a third type characterized by right-sided clonic facial movements. All seizures were brief and lasted approximately 20 s.

At the age of two months, the patient was referred to our Department of Pediatric Neurology. The clinical evaluation revealed growth delay (weight 3400 g (-3 SD), height 58 cm (Pc 62), microcephaly (OFC 35, -2.8 SD), dysmorphic facial features (hypertelorism and epicanthus), no eye fixation, severe generalized hypotonia with decreased spontaneous movements, and global developmental delay (i.e., a mental age of under one month).

The patient continued to present with seizures, for which he received multiple anti-seizure medications, such as phenobarbital, levetiracetam, and valproic acid, but with no seizure control. The patient suffered from recurrent respiratory infections; therefore, he did not benefit from adrenocorticotropic hormone treatment for seizure control. The ketogenic diet was refused by the family.

At the last evaluation, the patient presented with two types of seizures: the first type with fixed gaze and oral automatisms, lasting approximately 20 s, and the second type with bilateral clonic movements, lasting for seconds, and sometimes following the first type of seizure. EEG showed a pattern of alternating periods of hypsarrhythmia with suppression bursts. The child also had feeding difficulties with failure to thrive, and the morphometric parameters were as follows: weight of 11 kg (Pc 1, − 2.2 SD), height of 82 cm (Pc 2, -2.1 SD), and an occipitofrontal circumference of 42 cm (-4.5 SD). Neurological examination showed central hypotonia and profound global developmental delay, which is consistent with the developmental age of a pathological newborn.

### Brain MRI

**Fig. 1:**
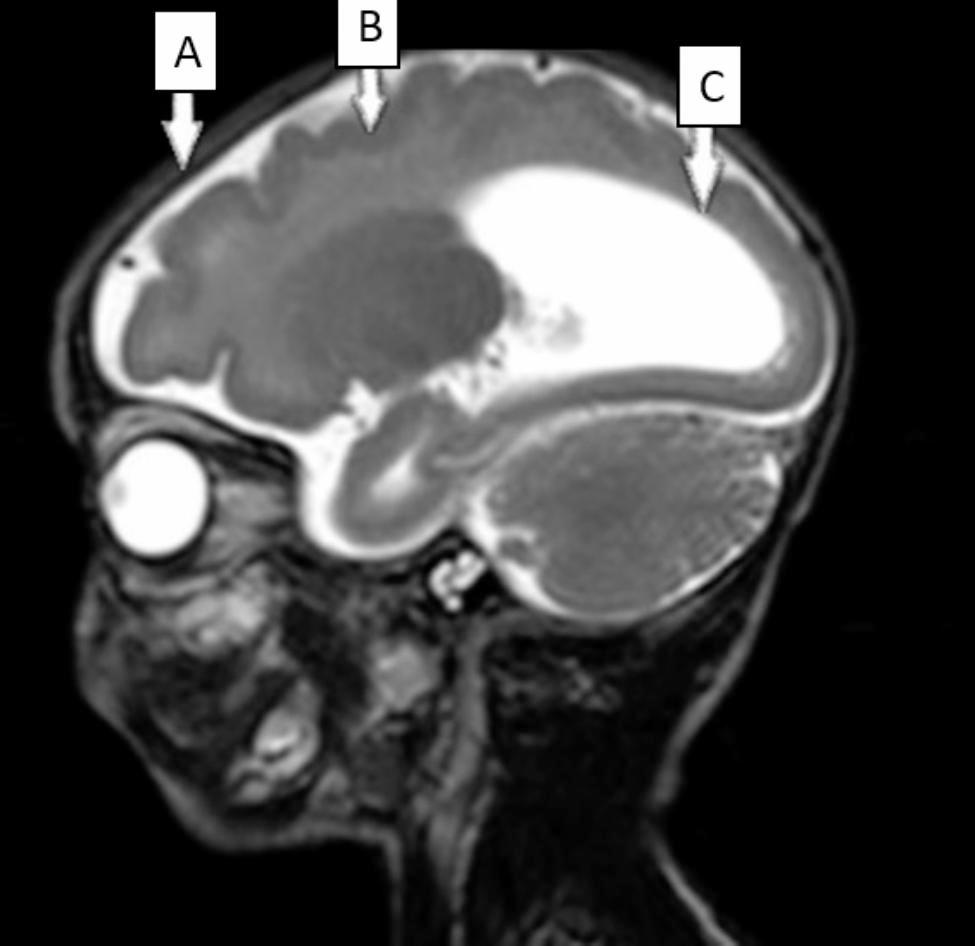
MRI results in our patient showing **(A)** superior frontoparietal lissencephaly, **(B)** subcortical band heterotopia (double-cortex), **(C)** enlargement of occipitotemporal lateral ventricles

1.5T MRI revealed superior frontoparietal lissencephaly, subcortical band heterotopia (double-cortex), partial agenesis of the corpus callosum with filiform genum, and bilateral enlargement of the occipitotemporal lateral ventricles (colpocephaly) (Fig. 1).

### Genetic testing results

Peripheral blood genomic DNA (gDNA) was isolated using the PureLink™ Genomic DNA Mini Kit (ThermoFisher Scientfic, Waltham, MA) according to the manufacturer’s protocol. Array-based comparative genomic hybridization (array-CGH) was performed using the CytoSure Constitutional Kit, version 3, 4 × 180k (Oxford Gene Technology) by following the manufacturer’s recommendations. NGS with an Epilepsy Panel comprising 308 genes was then performed for the proband in a private laboratory (Invitae Corporation, San Francisco, CA), as previously described [26].

NGS testing of the child, performed at the Invitae laboratory, revealed two deleterious variants in the *CCDC88A* gene (NM_001135597.1; NP_001129069.1): a single nucleotide deletion located in exon 15, leading to a stop codon at position 648 (c.1942del, p.Ile648Ter), and a duplication of two nucleotides in exon 24, leading to a frame-shift with a stop codon located 12 nucleotides downstream the duplication (c.4158_4159dup, p.Pro1387LeufsTer12). The variants were submitted to ClinVar by Invitae and made public under accession numbers VCV001365714.3 and VCV001435609.3 (https://www.ncbi.nlm.nih.gov/clinvar/variation/ VCV001365714.3 and https://www.ncbi.nlm.nih.gov/clinvar/variation/VCV001435609.3, accessed Jan. 26, 2024).

Sanger sequencing was performed for the entire *DCX* gene and the *CCDC88A* sequence variant evaluation. Variant screening of the coding sequences and exon-intron boundaries of *DCX* was performed as described by Papuc et al. [21]. *CCDC88A* direct sequencing was used for variant confirmation in the proband and inheritance model assessment in the parents. The *CCDC88A* primer design was developed using Primer3web version 4.1.0 (https://primer3.ut.ee/). One primer set (forward − 5’AGTTTTCCTCATCAAGTTGGGA3’ and reverse − 5’AGGAGAACGAGCTGAAGAACT3’) amplified a 242 bp fragment targeting the p.Ile648Ter variant; the other set (forward − 5’TCCAGTTGCCTCTCCTAACA3’, and reverse − 5’GCTGTACAAAATATGATGCCTGT3’) amplified a 294 bp fragment for p.Pro1387LeufsTer12 variant detection. These primers were used for both the amplicon generation and sequencing reactions. Polymerase chain reaction (PCR) was performed using a ProFlex 96 well thermocycler (ThermoFisher Scientific) in a total volume of 25 µl, which contained 2.5 µl reaction buffer 10X, 0.75 µl MgCl2 (50 mM), 0.5 µl dNTP (10 mM), 0.5 µl of each primer (10 µM), 0.1 µl Invitrogen Taq Polymerase Recombinant (5 U/µl) (ThermoFisher Scientific), nuclease free water, and 50 ng gDNA. The PCR program included an initial denaturation step (94 °C, 3 min), 35 cycles of denaturation (94 °C, 45 s), primer annealing (56 °C, 30 s), elongation (72 °C, 1 min), and a final elongation (72 °C, 5 min). PCR amplicons were purified with ExoSap-IT PCR Product Cleanup Reagent, and the BigDye Terminator v3.1 Cycle sequencing kit was used according to the manufacturer’s protocol (ThermoFisher Scientific). The forward and reverse products were sequenced on an ABI 3500 Genetic Analyzer (Applied Biosystems, Foster City, CA, USA). Sequence quality was assessed using Sequencing Analysis software (SeqA6) (ThermoFisher Scientific), followed by a comparative analysis of the patient data and the wild-type *CCDC88A* sequence (RefSeq NM_001135597.1).

**Fig. 2:**
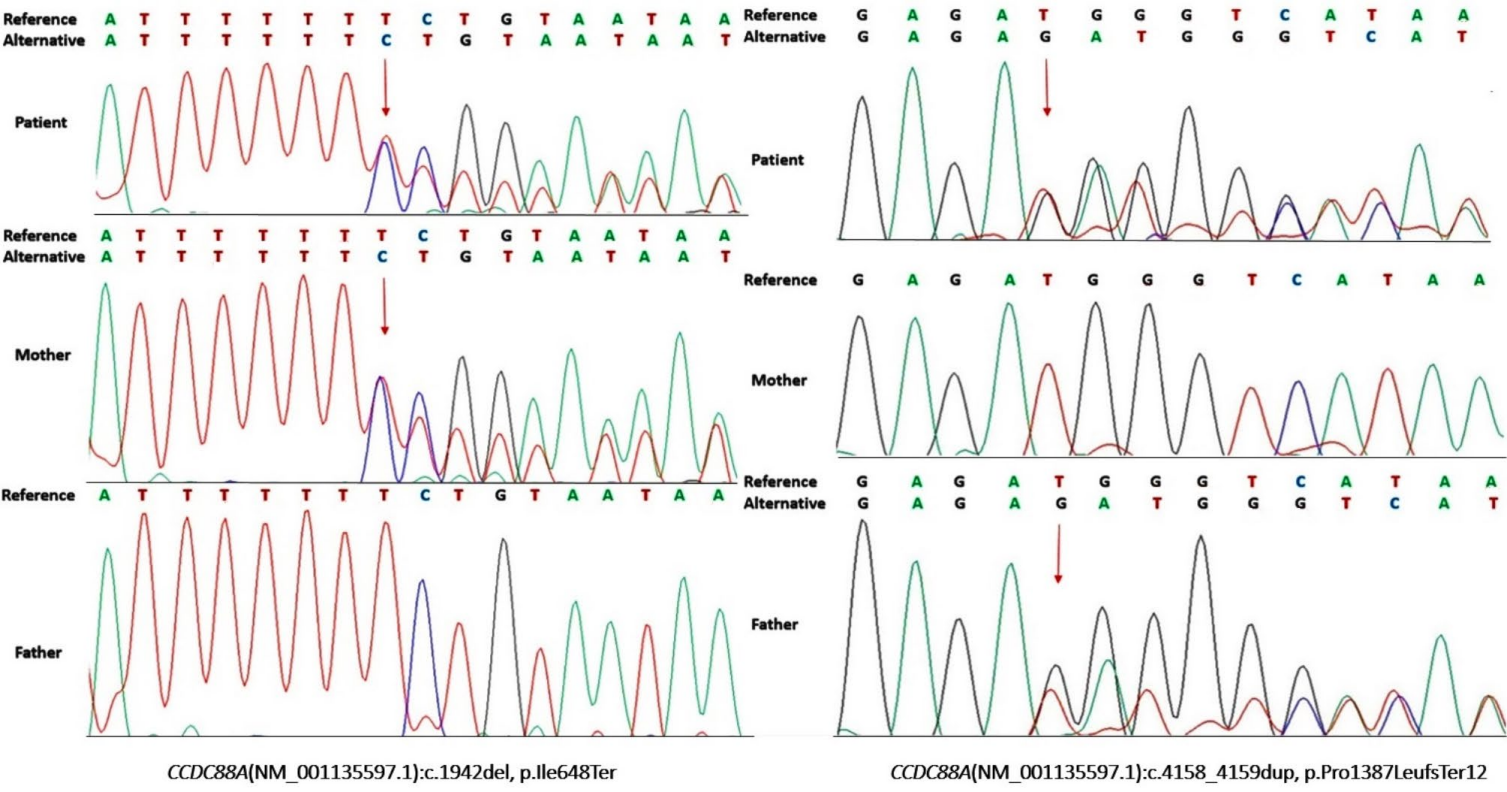
Sanger sequencing electropherograms showing the *CCDC88A* variants found in our patient and their inheritance pattern, confirming the biallelic disruption of the gene

The *DCX* gene direct sequencing and array-CGH revealed no variants with known pathogenic or uncertain significance. The Sanger sequencing confirmed the presence of the *CCDC88A* variants in the proband and parents. It was determined that p.Ile648Ter was maternally inherited, while p.Pro1387LeufsTer12 was inherited from the father, thereby confirming the *trans* configuration of the *CCDC88A* variants (Fig. 2).

This study was approved by the Ethics Committee at each of the institutions where the study took place. Written informed consent for performing all the necessary investigations, participation in the study, and data publication was obtained from the parents of the patient.

## Discussion and conclusions

We report on a complex clinical and imaging phenotype, including a neuronal migration defect, SBH, in a child harboring a newly discovered compound heterozygous pathogenic variant in the *CCDC88A* gene. The phenotype includes profound global developmental delay, congenital microcephaly, dysmorphic features, hypotonia, and epileptic encephalopathy.

Biallelic truncating variants in *CCDC88A* have been described in patients with progressive encephalopathy with edema, hypsarrhythmia, and optic atrophy (PEHO-like syndrome, MIM 617507). This type of EE caused by *CCDC88A* pathogenic variants is extremely rare. To date, only three reports have been published on this condition: one by Nahorski et al. [18], which included three patients from the same family, one by Abdulkareem et al. (2018) describing two siblings, and, recently, one child described by Issa et al. [10] (Table 1). All the children were born from consanguineous parents. Many clinical features were shared by all the reported patients, such as microcephaly, neonatal hypotonia, seizures, profound developmental delay, face and limb edema, and dysmorphic features, with a similar appearance of eyes, nose, mouth, and fingers (Tables 1 and 2). Regarding visual impairment, all patients exhibited poor or absent visual fixation, and optical atrophy was observed in five of the six patients. Our patient’s phenotype overlaps with most of these clinical traits, except for edema and optic nerve atrophy. EEG studies revealed hypsarrhythmia in the patients reported by Nahorsky et al. (2016) and Issa et al. [10], as well as in our patient.

**Table 1:** Clinical characteristics and genetic findings of our patient in comparison with previously reported patients with *CCDC88A* variants

Patient	GDD with absence of psychomotor milestones	Epilepsy	Hypotonia	Microcephaly	Dysmorphic features	Brain malformation	CCDC88A variants			
							Variant position (DNA, protein)	Zygosity status	Exon	Protein domain
**Our case**	**+**	**+**	**+**	**+**	**+**	**+**	**c.1942del**,** p.Ile648Ter** **c.4158_4159dup**,** p.Pro1387LeufsTer12**	**Het** **Het**	**E15** **E24**	**Coiled-coil** **Membrane binding**
Patient 1 (Nahorski et al.)	+	+	+	+	+	+	c.2313delT (p.Leu772Ter)	Ho	E15	Coiled-coil
Patient 2 (Nahorski et al.)	+	+	+	+	+	+	c.2313delT (p.Leu772Ter)	Ho	E15	Coiled-coil
Patient 3 (Nahorski et al.)	+	+	+	+	+	+	c.2313delT (p.Leu772Ter)	Ho	E15	Coiled-coil
Patients 4 (Abdulkareem et al.)	+	+	+	+	+	+	c.1292G > A (p.Trp431Ter)	Ho	E12	Coiled-coil
Patients 5 (Abdulkareem et al.)	+	+	+	+	+	+	c.1292G > A (p.Trp431Ter)	Ho	E12	Coiled-coil
Patient 6 (Issa et al.)	+	_+_	+	+	+	+	c.1795_1798del (p.Thr599ValfsTe4)	Ho	E15	Coiled-coil

GDD – global developmental delay; Het – heterozygous; Ho – homozygous; E - exon

Various brain malformations have been described in patients with *CCDC88A* variants. All three cases reported by Nahorski et al. [18] included pachygyria, polymicrogyria prominent in the Sylvian fissures, dilated ventricles, hypoplastic corpus callosum, subependymal cysts, and hypoplastic pons. The patient described by Issa et al. [10] also presented with MRI abnormalities, such as abnormal gyration with minimal augmentation of the cortical thickness, dilated ventricles, hypogenesis of the corpus callosum, demyelination, colpocephaly, and prominent basal ganglia. For the two siblings reported by Abdulkareem et al. (2018), brain atrophy was the only MRI feature described.

**Table 2:** Characteristics of the epileptic seizures in our patient and previously reported patients with *CCDC88A* variants

Patient	Onset in the first weeks of life	Seizures type			EEG anomalies/ pattern	Response to AEDs
		Complex focal seizures	generalized tonic-clonic seizures	Infantile spasms		
Our case	+	+	+	+	+ / Hypsarrhythmia	-
Patient 1 [18]	+	+	+	+	+ / Hypsarrhythmia	-
Patient 2 [18]	+	+	+	+	+ / Hypsarrhythmia	-
Patient 3 [18]	+	+	+	+	+ / Hypsarrhythmia	-
Patients 4 and 5 (Abdulkareem et al. 2018)	+	NA	NA	NA	NA	-
Patient 6 [10]	second month	-	+	+	+ / Generalized epileptogenic discharges	-

Legend: electroencephalogram (EEG); antiepileptic drugs (AEDs); not available (NA);

MRI of our patient showed brain malformations that were consistent in some of those previously reported, with the overlapping features including corpus callosum anomalies, abnormal gyration patterns, and colpocephaly. In addition, our patient presented with subcortical band heterotopia, a severe brain malformation that, until the present time, was not described in patients with PEHO-like syndrome and *CCDC88A* pathogenic variants.

**Fig. 3:**
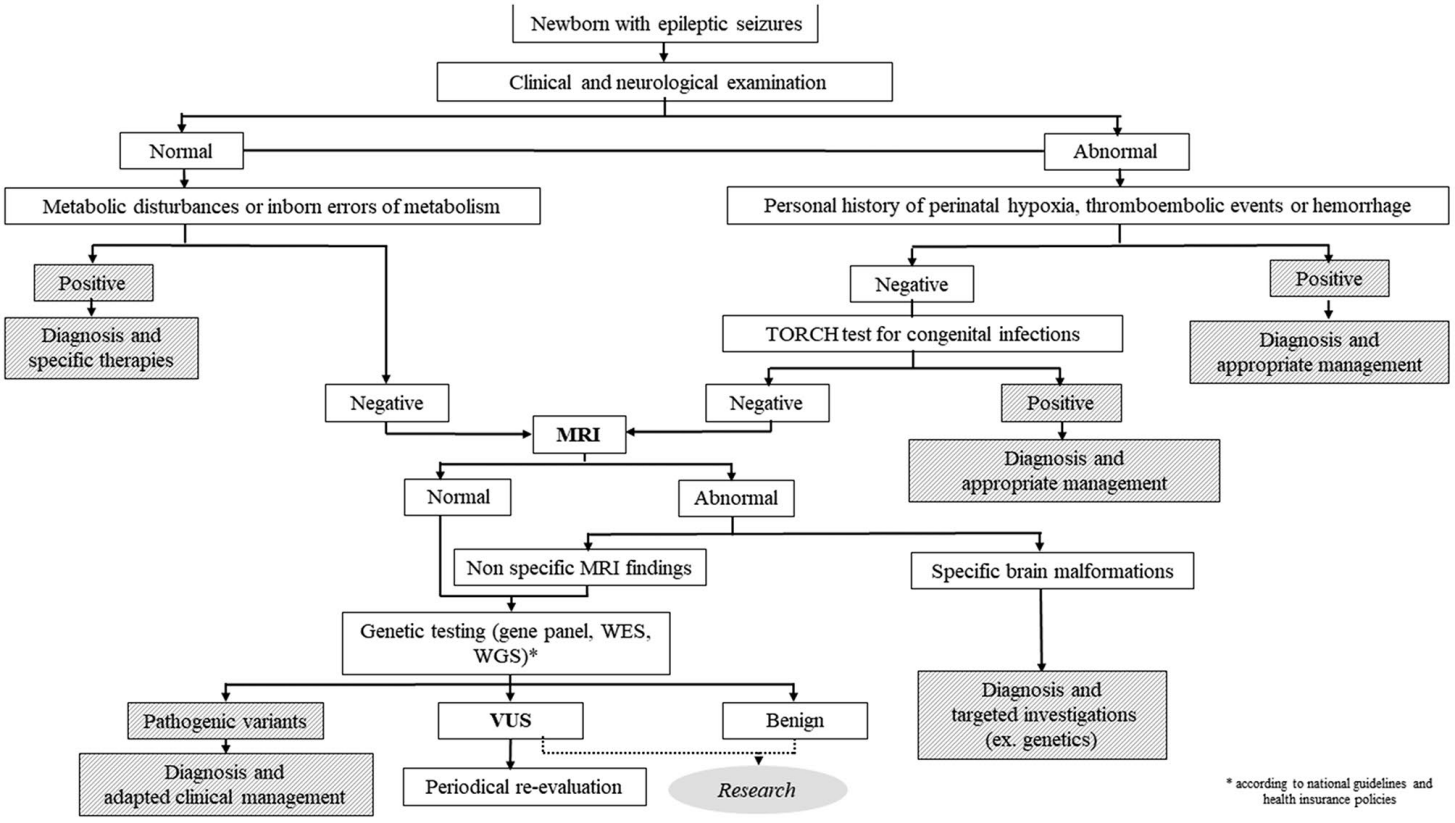
Diagnostic workflow of developmental and epileptic encephalopathies with early onset

The differential diagnosis of PEHO-like syndrome should include all types of developmental and epileptic encephalopathies with neonatal onset, such as early-infantile developmental and epileptic encephalopathy and infantile epileptic spasms syndrome. In most of the cases the etiology is genetic, with a wide range of genetic changes reported, from pathogenic sequence variants in genes such as *SCN2A*,* SCN8A*,* KCNQ2*,* TSC1*,* TSC2*,* ARX*,* STXBP1*,* CDKL5* to entire chromosome anomalies (e.g. trisomy 21). However, non-genetic causes should also be excluded, with emphasis on congenital infections (excluded based on specific blood tests for TORCH infections), metabolic conditions (no brain malformations are present on MRI), hypoxic ischemic encephalopathy (excluded based on child history for hypoxia at birth and on specific brain MRI lesions) [29]. The diagrammatic representation of a proposed diagnostic workflow can be found in Fig. 3.

The severity of the clinical findings observed in all the patients indicates the biological significance of *CCDC88A*, specifically in brain development. The *CCDC88A* gene encodes a protein, also known as Girdin, which is involved in various biological processes, such as cell, organ, and embryo development [3, 11, 23, 28], neuronal and tumoral cell migration [4, 6, 8, 9, 12, 20, 22, 27], and cancer cells invasion and metastasis [5, 7, 12]. Girdin is expressed across all human tissues, with the highest level being recorded in the brain and testis [4, 17].

The Girdin protein encompasses 1870 aa and has a complex domain architecture that comprises an N-terminal (NT) end represented by a microtubule-binding Hook domain that binds DISC1 (1-196 aa) [24], a coiled-coil domain (196–1304 aa) that plays a role in homodimerization [4, 5], and a terminal (CT) region that includes domains and sites relevant to Girdin’s function. The first of the CT domains, the Gά-binding domain (GBD) (1343–1424 aa), encompasses a PI4P-binding site (1390–1408), which mediates interactions with the plasma membrane and Golgi apparatus, as well as other possible unknown functions. Next to the PI4P-binding site stands the phosphorylation site of AKT serine/threonine kinase 1 (AKT1) (Serine 1417). The terminal part of the CT region contains amino acids 1623–1870 and is responsible for interactions with the actin filaments, AKT1, and epidermal growth factor receptor [4, 8].

The *CCDC88A* pathogenic variants described previously [1, 10, 18] are homozygous, truncating variants located in the coiled-coil domain of the protein. Our patient presented with truncating variants in a compound heterozygous pattern, one of which was located in the coiled-coil domain and the other affecting GBD. All of these variants, including the variants in our patient, have been predicted to generate truncated proteins, as they lack the CT domains. Nahorski et al. [18] performed gene expression studies and demonstrated that mRNA containing the variant was present in a proband’s blood sample and was thus not degraded by nonsense-mediated decay. However, the expression level was lower in the patient than in his parents. The production of a truncated protein was found by cloning studies, suggesting that this is the most likely mechanism of the disease [18]. These results further imply that the CT region of *CCDC88A* is critical for the normal function of Girdin. Mouse studies [2] have shown that Ccdc88a knockout animals exhibit mesiotemporal lobe epilepsy and postnatal growth retardation with early-age lethality. The same results were observed by Nahorski et al. [18], and further analysis of brain anatomy in Ccdc88a knockout mice revealed microcephaly and corpus callosum developmental anomalies, which partially reflected the human brain phenotype.

The above findings indicate that *CCDC88A* may be a critical gene for normal neurodevelopment that impacts both the function and the anatomical structure of the mammalian brain [16, 18]. Several studies have revealed that Girdin interacts with the Disrupted-In-Schizophrenia 1 protein (DISC1), an important regulator of neuronal migration and differentiation during mammalian brain development from embryonic stages to adulthood [6, 14, 15]. Using curated experimental data from the existing literature and applying a mathematical model (Boolean network), John et al. [13] detailed the DISC1 interactome involved in the regulation of neuronal migration. DISC1 interacts with 18 proteins, which were categorized into eight functional modules. In this system, Girdin, together with AKT1 and actin beta, forms a distinct functional module that mediates the tangential migration of cortical interneurons [13, 25]. It should be noted that in an earlier study, Enomoto et al. [4] proved for the first time that Girdin, an actin-binding protein that is phosphorylated by Akt1, is essential for actin cytoskeleton organization and cell migration. Furthermore, Girdin was found to be involved in the migration of new neurons from the ventricular–subventricular zone of the lateral ventricles to the olfactory bulb, thereby contributing to the development of the postnatal mouse brain [20, 27].

The *CCDC88A* gene may also play an important role in primary cilia development and function. For instance, Nechipurenko et al. [19] demonstrated that Girdin regulates cilia morphology by positioning the basal body for cilium formation in the sensory neurons of *C. elegans* and human RPE-1 cells [19]. Future studies investigating the disruption of Girdin might provide new data on its involvement in central nervous system development, thus helping to explain human structural brain anomalies in patients with truncating variants of *CCDC88A*.

In conclusion, we present a patient with a PEHO-like clinical picture harboring a novel sequence variant of the *CCDC88A* gene, thus contributing to the phenotypic and genotypic delineation of *CCDC88A*-related EEs. In addition, our patient presented with a complex brain malformation, including subcortical band heterotopia, which was not reported in previous cases.

## Data Availability

The data that support the findings of this study are available from the corresponding author on reasonable request.

## Abbreviations

AED: Antiepileptic drug
AKT1: AKT serine/threonine kinase 1
array: CGH-array based comparative genomic hybridization
CCDC88A: Coiled-Coil Domain-Containing Protein 88 A
CT: C-terminal
DISC1: Disrupted-in-Schizophrenia 1 protein
E: Exon
EEs: Epileptic encephalopathies
EEG: Electroencephalogram
GBD: Gά-binding domain
GDD: Global developmental delay
gDNA: genomic DNA
Het: Heterozygous
Ho: homozygous
NA: Not available
NGS: Next generation sequencing
NT: N-terminal
PCR: Polymerase chain reaction
PEHO: Progressive encephalopathy with edema, hypsarrhythmia and optic atrophy
